# Behavioural Response Thresholds in New Zealand Crab Megalopae to Ambient Underwater Sound

**DOI:** 10.1371/journal.pone.0028572

**Published:** 2011-12-07

**Authors:** Jenni A. Stanley, Craig A. Radford, Andrew G. Jeffs

**Affiliations:** Leigh Marine Laboratory, University of Auckland, Warkworth, New Zealand; University of Southern California, United States of America

## Abstract

A small number of studies have demonstrated that settlement stage decapod crustaceans are able to detect and exhibit swimming, settlement and metamorphosis responses to ambient underwater sound emanating from coastal reefs. However, the intensity of the acoustic cue required to initiate the settlement and metamorphosis response, and therefore the potential range over which this acoustic cue may operate, is not known. The current study determined the behavioural response thresholds of four species of New Zealand brachyuran crab megalopae by exposing them to different intensity levels of broadcast reef sound recorded from their preferred settlement habitat and from an unfavourable settlement habitat. Megalopae of the rocky-reef crab, *Leptograpsus variegatus*, exhibited the lowest behavioural response threshold (highest sensitivity), with a significant reduction in time to metamorphosis (TTM) when exposed to underwater reef sound with an intensity of 90 dB re 1 µPa and greater (100, 126 and 135 dB re 1 µPa). Megalopae of the mud crab, *Austrohelice crassa*, which settle in soft sediment habitats, exhibited no response to any of the underwater reef sound levels. All reef associated species exposed to sound levels from an unfavourable settlement habitat showed no significant change in TTM, even at intensities that were similar to their preferred reef sound for which reductions in TTM were observed. These results indicated that megalopae were able to discern and respond selectively to habitat-specific acoustic cues. The settlement and metamorphosis behavioural response thresholds to levels of underwater reef sound determined in the current study of four species of crabs, enables preliminary estimation of the spatial range at which an acoustic settlement cue may be operating, from 5 m to 40 km depending on the species. Overall, these results indicate that underwater sound is likely to play a major role in influencing the spatial patterns of settlement of coastal crab species.

## Introduction

Crabs are important species, ecologically in many coastal communities, often playing a significant role as active benthic predators and in physically altering habitats through burrowing. Crab fisheries are also commercially important, with approximately 1.34 million tonnes harvested in 2009 [1]. Most crab species have a pelagic larval stage which can last weeks to months depending on the species, before the larvae settle into benthic habitats and metamorphose to a benthic dwelling juvenile [2]. Despite the widespread importance of crabs, their settlement and recruitment biology is relatively poorly understood, even though it is likely to play a major role in determining the size of the subsequent population.

A number of settlement cues, including chemical, physical and acoustic cues, have been identified that have shown to be involved in assisting pelagic larval stages of marine organisms to locate and settle into suitable benthic habitats [3], [4], [5], [6]. Previously studied settlement cues in crabs, such as chemical and tactile cues, have their limitations, as tactile cues are thought to be only effective at very fine spatial scales [7] and chemical stimuli are carried by water currents meaning that they can only be effective either downstream of the source, or at small distances before they become greatly diluted [8]. Ambient underwater sound has been long regarded as one of the most probable cues for guiding onshore orientation by pelagic larvae [9], [10], [11]. A small number of studies have shown that underwater sound emanating from coastal reefs can strongly influence the swimming direction, initiate settlement behaviour and greatly advance the physiological development in settlement stage crab larvae [12], [13], [14]. Therefore, it is likely that underwater sound may be of considerable ecological importance in influencing the settlement success of coastal crustaceans. However, there has been no investigation into the behavioural response thresholds of settlement stage crabs to different levels of intensity of underwater sound they may encounter in the marine environment. Knowledge of their behavioural response thresholds can then be used to provide some initial estimates of the spatial scale on the coast over which an acoustic cue could operate for the settlement stage crab larvae based on well-described theoretical underwater acoustic transmission loss models [15].

Compared to the abundance of knowledge concerning the visual, tactile and chemosensory systems in decapod crustaceans, the acoustic sensory systems in these animals remain relatively unknown [16]. However, to date there have been a few studies that have shown clear behavioural responses to underwater auditory cues in late-stage larval crabs [12], [13], [17]. Using an *in*-situ binary choice chamber coupled with an artificial source of underwater reef sound, the megalopae of all five crab species tested showed a significant positive swimming response towards the sound source [13]. In another recent study the megalopae of five crab species showed marked changes in swimming behaviour and a significant decrease in time to metamorphosis (TTM) when exposed to replayed underwater reef sounds at ambient levels than when compared to a silent (control) treatment [12]. The megalopae exposed to sound decreased swimming activity earlier and exhibited crawling behaviour that was a precursor to settlement and metamorphosis, and also decreased the median TTM by almost half in some species. During a similar study TTM was significantly reduced when megalopae of five reef brachyuran species were exposed to ambient underwater sound recorded at a preferred habitat type (rocky reef or coral reef) compared with ambient sound from an unfavourable habitat type (sandy beach or lagoon) [17]. These responses were only elicited in megalopae upon exposure to the reef sound, whereas underwater sound from other habitat types, such as an open sandy beach, failed to produce a settlement and metamorphosis response. These consistent results occurring in both temperate and tropical crab species indicated that the phenomenon has the potential to be widespread geographically among reef dwelling species of brachyuran crab [12]. Furthermore, the consistency of the results in both laboratory and field experiments and among various species, also show the experimental protocol used in these studies is capable of producing reliable results [17]. Therefore, the aim of the current research was to determine the behavioural response thresholds of four species of New Zealand crab megalopae by experimentally exposing them to different levels of broadcast reef sound recorded from their preferred settlement habitat, and from an unfavourable settlement habitat. The determination of the acoustic behavioural response thresholds from this research can be used to estimate the spatial range over which crab megalopae should be capable of using an acoustic settlement cue.

## Methods

The study was undertaken during October 2010 to December 2010 in temperate waters near the Leigh Marine Laboratory in north-eastern New Zealand.

### Ethics Statement

The work was conducted under University of Auckland Animal Ethics Committee approval number R701.

### Source of megalopae

Light traps were used to capture pelagic megalopae for the behavioural response threshold experiments [18], [19]. Up to four light traps were deployed on dusk within 500 m of the shoreline, 7–30 m apart, dependant on the deployment location, and submerged 2 m from the surface in water of 5–10 m depth. The traps were recovered within 2 hours of sunrise the following morning. When large planktivorous fishes were found in a light trap, megalopae were not used for experimentation as they may have altered behaviour due to stress from being in the presence of a predator [20]. The megalopae were transported in seawater to the nearby Leigh Marine Laboratory where they were counted, sorted by developmental stage and the species identified. Only intermoult pre-settlement (i.e., natant and active swimming) megalopae of a similar size and age were selected for use in the experiments. Suitable species of megalopae were held in a flowing filtered (40 µm) seawater system with natural light period and ambient temperature (15–22°C, dependant on timing) until experiments begun the following evening. Four species of temperate brachyuran megalopae were used. *Hemigrapsus sexdentatus*, *Cyclograpsus lavauxi* and *Leptograpsus variegatus* are all common coastal species of crabs in New Zealand that are from the family Grapsidae. The adults of these species are known to be associated with nearshore subtidal and intertidal habitats, most often living under boulders, amongst macroalgae and on rocky shores [21]. *Austrohelice crassa* is also from the family Grapsidae, however, adults are known to be associated with enclosed beaches, sheltered harbours, lagoons, estuaries, and mangrove swamps [22]. Individuals of this species will usually construct a burrow in consolidated benthic sediment [21].

### Sound recordings for threshold experiments

Recordings of the typical ambient underwater sound were made at two different shallow water habitats (i.e., a macroalgae dominated rocky reef and an open sandy beach) for use in the behavioural threshold experiments. Sound treatments were recorded from north-eastern New Zealand during the summer at dusk on a new moon; North Reef (36°15′54.14″S, 174°47′37.47″E) a macroalgae dominated rocky reef and Pakiri Beach (36°13′33.85″S, 174°42′31.96″E) an open sandy beach. *In situ* habitat sounds were recorded using a hydrophone hanging beneath a float to eliminate extraneous noise associated with recording directly from a floating vessel. The recording system consisted of a calibrated HTI-96-MIN wideband and omnidirectional hydrophone (High Tech, Inc., flat frequency response over the range of 10–24,000 Hz) that was weighed down vertically to 10 m water depth and suspended from the outside of a sealed floating barrel which contained a Sound Devices, LLC. −722 solid state recorder (48 kHz; 24-bit). Several 5 min recordings were taken at 1700–1800 h (dusk) in approximately 15–20 m of water at each habitat site at about 20 m from the margin of the coastal fringing reef at the reef site and 100 m from the shoreline at the sandy beach site. No anthropogenic sources of noise, such as large ships or power boats, were present in the vicinity at the time of recording. All recordings were conducted in near calm conditions (<0.5 m wave height and <2.6 ms^−1^ wind speed) (Climate Station, Leigh Marine Laboratory). Digital recordings from the recorder were transferred to a PC and analysed using MATLAB software (The MathWorks, Inc.) with codes specifically written for the recordings to calculate sound levels and produce power spectra.

### Laboratory-based threshold experiments

Each laboratory-based experiment consisted of five sound treatments (four distinct sound levels and one silent), and within each treatment there were three replicate water baths used to maintain a constant water temperature for megalopae throughout the experiment. The baths were acoustically isolated using rubber mats to prevent any transfer of acoustic energy from the surrounding environment into the experimental treatments. The absence of any significant acoustic signal in the Silent treatment tanks was confirmed by recording with a calibrated hydrophone (High Tech, In. HTI – 96 – MIN) and determining the sound level of any recorded sound.

Each replicate water bath contained 5–10 plastic vials (250 ml) with a sealed lid housing a single randomly selected megalopa in filtered (1 µm) and UV treated seawater. The vials had a roughened base acting as a chemically inert settlement surface for the megalopae. All replicates for both the sound treatments and Silent treatment had a weighted Phillips loudspeaker (4 Ω, 5 watts) inside a watertight plastic bag which was submerged in the water bath. For the sound replicates only, a Sony CD Walkman D – EJ815 was connected to the speaker and used to continually play a 4 min loop of recorded ambient underwater reef sound into the water bath and through the acoustically transparent plastic containers holding the crabs.

When on a single night sufficient (>150) megalopae of the same species were collected from the light traps to conduct the experiments, the crabs were randomly allocated to an experimental treatment and replicate. All megalopae in each treatment were kept under natural light period and ambient water temperature (15–22°C, depending on local ambient temperature) for the duration of the experiment. All laboratory-based experiments were conducted in a quiet laboratory with restricted access.

The megalopae were added to the experiment at 1700 h on the day of their capture and the CD Walkman was switched on to initiate sound in the sound treatments. Subsequently every 6 h an observational period occurred, at which time counts were made of the number of megalopae that had settled onto the base of the vials and metamorphosed into the first instar benthic juvenile stage. The time from establishing the experiment to the first observational period when a first instar juvenile was observed was termed the time to metamorphosis (TTM). Each period of observation lasted no more than 40 min for all treatments. When the observational period occurred at night, pale red light was used to observe megalopae behaviour because prior testing demonstrated there was little or no visual response by megalopae to the red lighting [23]. In this study ‘settlement’ is defined as a behavioural process which involves movement out of the water column to a benthic substrate, and ‘metamorphosis’ as a physiological process which includes loss of larval characteristics retained in the megalopa and the completion of the moult to the reptant body form of a juvenile crab [24]. A behavioural response threshold was determined by the lowest sound level for which TTM was significantly shorter than the TTM for the Silent treatment.

The experiment was terminated when all experimental megalopae in all treatments had metamorphosed. The juvenile crabs were kept for 5–10 d following the experiment in flowing seawater, fed and monitored for post-experimental mortality.

### Tank set-up for North Reef and Pakiri Beach experiments

A calibrated hydrophone and recorder (High Tech, Inc., Mississippi, USA HTI – 96 – MIN, Sound Devices, LLC., Wisconsin, USA 722 recorder) was used to adjust the sound level produced by the loudspeakers in each experimental sound treatment tank. The sound levels generated by the digital recordings were adjusted to reach the desired level set for each experimental treatment using Adobe Audition software (Adobe Systems, Inc.).

Separate experiments were run for the two different habitat sounds (i.e., North Reef and Pakiri Beach), to determine the sound level at which crab megalopae demonstrated reduced TTM compared to the Silent treatment, i.e., the behavioural response threshold. However, it was not appropriate to make direct comparisons of median TTM values between the two separate experiments because the experiments were conducted with different cohorts of wild-caught megalopae that could have been at slightly different stages of development.

For the experiments using recorded sound from North Reef the following experimental sound level treatments were used; 135 dB re 1 µPa (High), 126 dB re 1 µPa (Ambient level – as determined from field recording), 100 dB re 1 µPa (Low), 90 dB re 1 µPa (Lowest) RMS level in the 100–24000 Hz range and Silent treatment (no replayed sound). For the experiments using recorded sound from Pakiri Beach the following sound level treatments were used; 125 dB re 1 µPa (High), 103 dB re 1 µPa (Ambient level – as determined from field recording), 90 dB re 1 µPa (Low) RMS level in the 100–24000 Hz range and Silent (no replayed sound). There was also an additional treatment included in this experiment; 126 dB re 1 µPa (Ambient Reef sound– as determined from field recordings at North Reef). This extra sound treatment was included to provide a direct comparison of the results from the Pakiri Beach sound treatments, with a sound cue from a preferred settlement habitat, i.e., North Reef habitat.

The replayed sounds in the experimental tanks were recorded with a calibrated hydrophone (High Tech, Inc., HTI – 96 – MIN) for comparison with the source signals recorded from the natural habitats and the spectral composition analysed using MATLAB software with codes specifically written for these recordings.

### Data analyses

For the experiments for each species, the non-parametric Kruskal-Wallis comparison of ranks was used to test for a difference in the median TTMs among the replicates within the same treatment (i.e., each treatment analysed separately), because the data was not continuous [25]. If this test found no difference among the three replicates, the data from the replicates were pooled for each treatment and then used in an experiment-wide comparison of treatments using the Kruskal-Wallis test to compare the median TTMs. For all statistical tests, *P* values≤0.05 were considered to be significant. To isolate differences among individual treatments a Dunn's pairwise multiple comparison procedure was used to test for differences among each treatment combination because not all sample sizes were equal. A metamorphosis rate for each treatment within each species was also calculated with a Sen's slope analysis for the data points between the last sampling event prior to the first megalopa metamorphosing and the sampling event when the last megalopa metamorphosed. A one way analysis of variance (ANOVA) was used to test for a difference in the mean metamorphosis rate among treatments using rates calculated for each replicate within treatments. Tukey's and Dunn's tests were used to test for differences among every treatment combination where the overall ANOVA was statistically significant. All analyses were performed using the software Sigma Stat 4.0 (Systat Software, Inc.) and Minitab 16.1.0 (Minitab, Pty.).

### Estimates of potential transmission range of acoustic settlement cue

The observed threshold levels determined in the different crabs species tested were used in conjunction with theoretical acoustic transmission loss models (spherical and cylindrical spreading from a point source) to estimate at what distance from the source (settlement habitat) the acoustic cue would be detectable by megalopae given the measured behavioural response thresholds [15]. For the recordings taken at 20 m from the reef an additional 13 dB was added to match the estimated source level at the reef based on calculations of cylindrical spreading from the reef source [15]. For the purposes of comparison it was assumed that megalopae would show a behavioural response to the sound once they were sufficiently close to the source habitat that the ambient sound level was the same as the threshold level (TL) for the crab. This assumption leads to the following equations for spherical and cylindrical spreading from measured level (ML) that were then used to estimate the range (R) at which the megalopae were theoretically able to respond to the underwater reef sound. However, these cylindrical spreading models are thought to be conservative for estimating the travel of reef sound [26]. Attenuation was not accounted for in the model as underwater sounds below 10 kHz lose less than 1 dB km^−1^ due to absorption by the medium [27].

Spherical spreading ML−A+13 = 20log (R)

Cylindrical spreading ML−A+13 = 10log (R)

## Results

### Sound analyses for North Reef experiment

**Figure 1:**
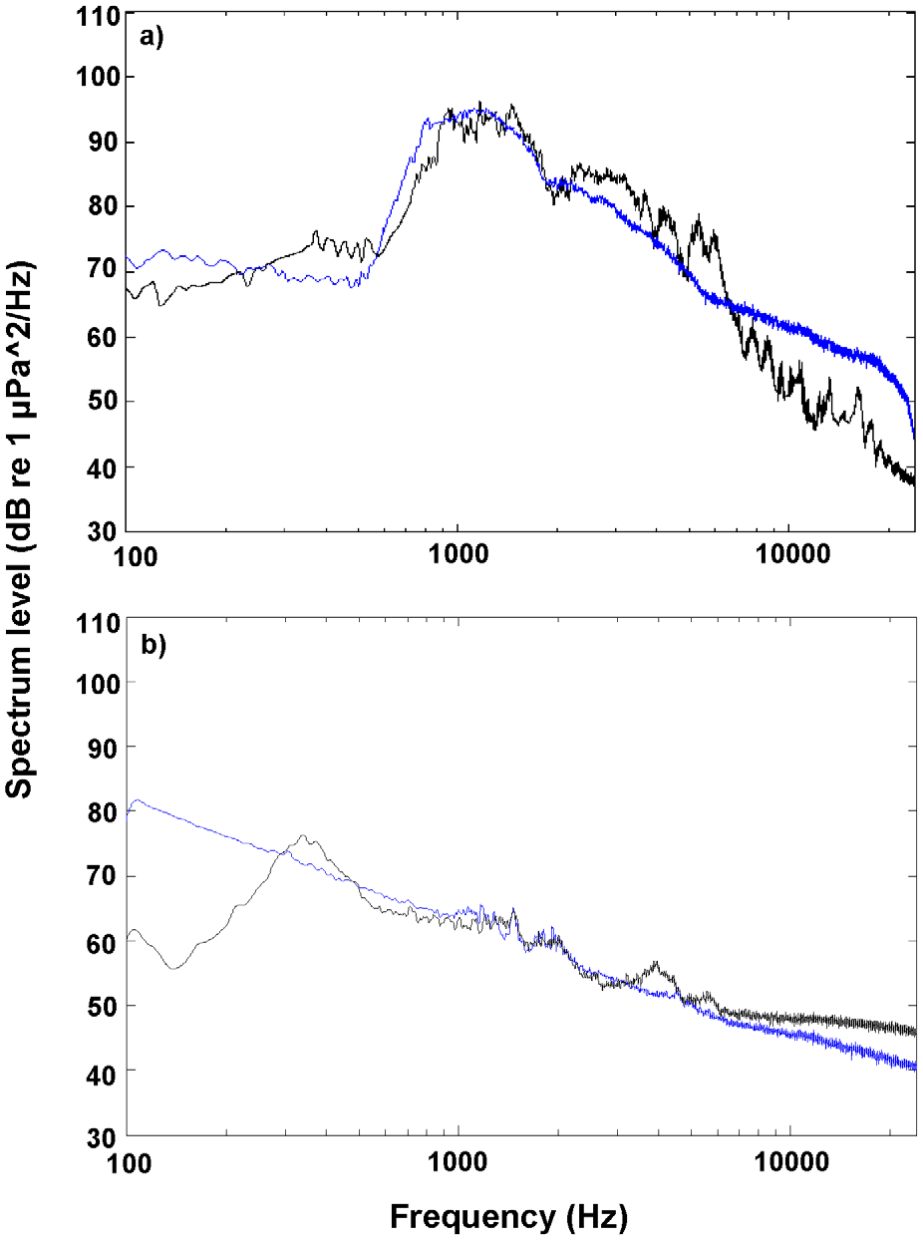
Spectral plots showing composition and sound level of ambient underwater sound. a) North Reef and, b) Pakiri Beach. Blue lines represent original natural ambient sound and black lines represent experimentally replayed sound.

The broadcast sound within the experimental tanks had a similar overall spectral composition to the source signals recorded from the natural habitat. In the original field recordings (North Reef) there was a peak in the spectra around 700–1200 Hz, which is produced by the feeding of the sea urchin, *Evechinus chloroticus*, whereas the higher frequency pulses were predominantly the snaps of snapping shrimp (Figure 1a). The power spectra of the experimental tanks showed that the frequency composition of the replayed habitat sound were reasonably consistent with the original field recording, with a small reduction in sound level in the higher frequencies (10000–24000 Hz) (Figure 1a). The Silent treatment had no sound transfer from any external sources. The flat response at approximately 34 dB represents the lower recording limit of the sound recording equipment.

### North Reef threshold experiments

In all four crab species that were tested there was no significant difference in the median TTM among the replicates within each of the five sound treatments (*P*>0.05). Therefore, for each species the TTM data for the replicates were pooled for each treatment to then test for an overall treatment effect.

**Figure 2:**
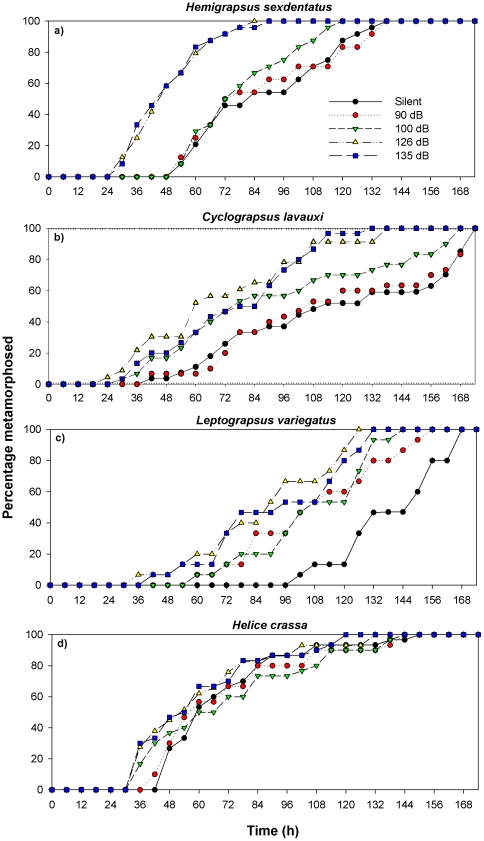
Settlement response plot showing percentage of megalopae metamorphosed over time (h) to various levels of North Reef sound. a) *Hemigrapsus sexdentatus* (n = 120), b) *Cyclograpsus lavauxi* (n = 140), c) *Leptograpsus variegatus* (n = 75), and d) *Austrohelice crassa* (n = 150).

Median TTM differed significantly among the sound treatments for the megalopae of all three rocky reef species tested; *H. sexdentatus* (Kruskal-Wallis test, H = 53.9, *P*<0.001), *C. lavauxi* (H = 25.8, *P*<0.001) and *L. variegatus* (H = 23.8, *P*<0.001) (Table S1, Figure 2a, b and c) with High and Ambient sound treatments consistently producing the shortest TTM. Using Tukey's pairwise multiple comparisons, *Hemigrapsus sexdentatus* had the most separation among the sound level treatments, with significant differences in median TTM identified between six of the ten pairs of treatment comparisons. *Cyclograpsus lavauxi* had significant differences in median TTM between eight of the ten treatment comparisons. *Leptograpsus variegatus* had significant differences in median TTM between four of the ten treatment comparisons. For all three species the Silent treatment consistently had the longest median TTM, and the Ambient sound treatment consistently had the shortest median TTM. *Leptograpsus variegatus* had the lowest behavioural response threshold of 90 dB, followed by *C. lavauxi* with a threshold of 100 dB, and lastly *H. sexdentatus* with 126 dB re 1 µPa. The median TTM did not differ significantly among the different sound level treatments of unfavourable settlement habitat (North Reef) for the tunnelling mud crab, *Austrohelice crassa* (H = 6.131, *P* = 0.177).

### Rates of metamorphosis in North Reef experiments

In the North Reef experiments two of the rocky reef species, *H. sexdentatus* and *C. lavauxi* both had significantly higher mean metamorphosis rates in the High and Ambient treatments than the Low, Lowest and Silent treatments (ANOVA, F = 5.8 & 11.2, *P* = 0.002 & 0.001 respectively, Table S1, Tukey's test *P*>0.05). The mean metamorphosis rate in *Cyclograpsus lavauxi* was 1.6 times faster in the High treatment than in the Silent treatment. *Leptograpsus variegatus and A. crassa* did not have increasing metamorphosis rates with increasing sound level.

### Estimates of potential detection range of acoustic settlement cue

Using the measured ambient sound levels recorded from North Reef it was estimated that megalopae of *L. variegatus* could be expected to show a settlement and metamorphosis behavioural response from the reef out to a distance of 199 m assuming spherical spreading, and out to 39811 m from the reef assuming cylindrical spreading. These estimated distances were considerably shorter for *C. lavauxi* because this species had a higher behavioural response threshold, out to 89 m and 7943 m assuming spherical and cylindrical spreading of the sound from the source respectively. *Hemigrapsus sexdentatus* had a higher behavioural threshold again with an estimated detection range between 5 m and 20 m from the source assuming spherical and cylindrical spreading of sound from the source respectively.

### Sound analyses for Pakiri Beach experiment

In the field recordings at Pakiri Beach the low frequencies in the range of 100–800 Hz were dominant, which is mostly likely due to abiotic noise sources (i.e., wind and waves) (Figure 1b). There were also low levels of higher frequency sound present, probably derived from distant reefs. The power spectra from the experimental playback tanks showed that the sound had a similar overall spectral composition to the original field recordings except for slightly reduced levels in the lower frequencies (100–300 Hz) for the Pakiri Beach High (125 dB re 1 µPa), Ambient (103 dB re 1 µPa) and Low (90 dB re 1 µPa) treatments (Figure 1b). This reduction in sound level in the lower frequencies is due to some limitation of the sound reproduction capabilities of the speakers used in the experiments. However, the composition of the higher frequencies (301–20000 Hz) remained fairly consistent with that of the field recordings, but with some slight variations due to the effects of replaying sound in small tanks (Figure 1b). In the Ambient Reef treatment there was a peak in the spectra around 700–1200 Hz, and higher frequency pulses from 200–10000 Hz. The Silent treatment had no sound transfer from any external sources.

### Pakiri Beach threshold experiments

In both crab species that were tested, *H. sexdentatus* and *L. variegatus*, there was no significant difference in the median TTM among the replicates within each of the five sound treatments (*P*>0.05). Therefore, for each species the TTM data for the replicates were pooled within each treatment to then test for an overall treatment effect.

**Figure 3:**
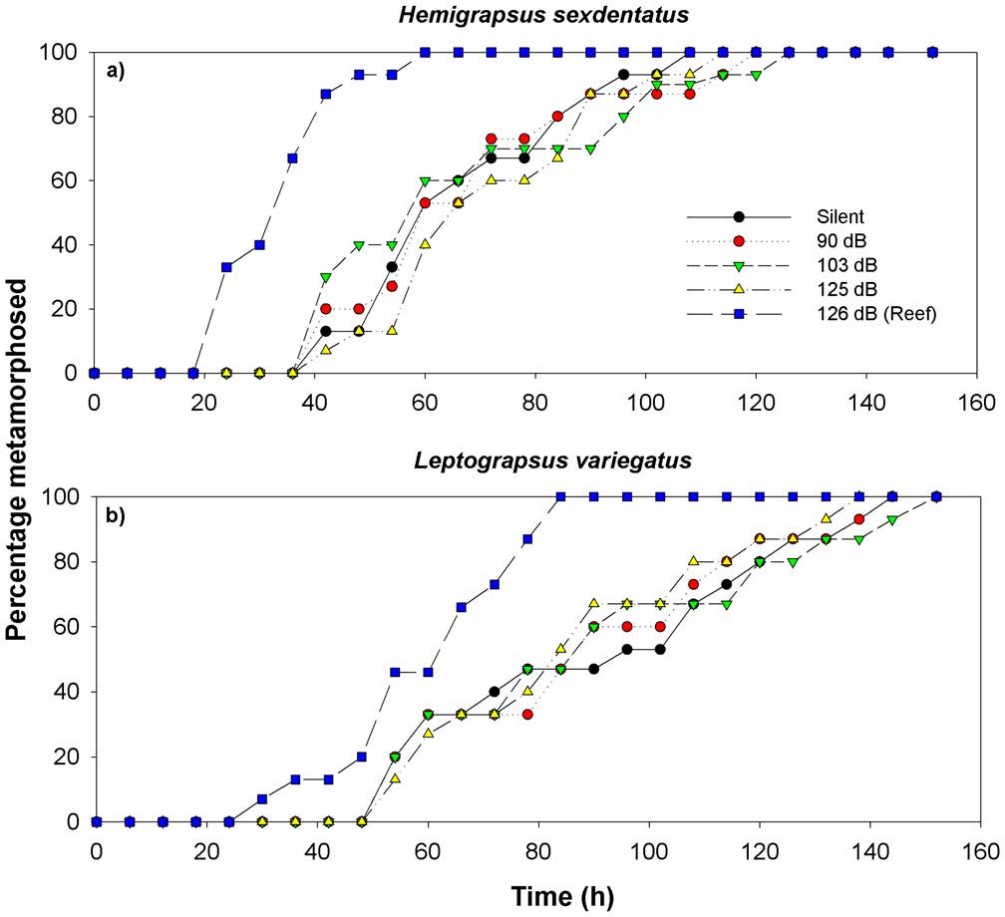
Settlement response plot showing percentage of megalopae metamorphosed over time (h) to various levels of Pakiri Beach sound. a) *Hemigrapsus sexdentatus* (n = 75) and *Austrohelice crassa* (n = 75).

Median TTM differed significantly among the sound treatments for the megalopae of both species tested; *H. sexdentatus* (Kruskal-Wallis test, H = 26.8, *P*<0.001) *A. crassa* (H = 12.8, *P*<0.001) (Table S2 & Figure 3a and b) with the Ambient Reef sound treatment consistently producing the shortest TTM when compared with the Pakiri Beach sound treatments at the three sound levels and a Silent treatment. Using a Dunn's pairwise multiple comparisons there was shown to be no significant difference in median TTM among the three Pakiri Beach sound level treatments (High 125 dB, Ambient 103 dB and Low 90 dB re 1 µPa) and the Silent treatment for both *H. sexdentatus* and *L. variegatus* (*P*>0.05). However, there was a significant difference between each Pakiri Beach sound level and the Ambient Reef sound treatment in both species.

### Rates of metamorphosis in Pakiri Beach experiment

In the Pakiri Beach experiments both species (*H. sexdentatus* and *L. variegatus*) had a significantly faster mean metamorphosis rate in the Ambient Reef sound treatment when compared to all of the other sound treatments (Pakiri Beach High, Ambient, Low and Silent) (ANOVA, F = 32.3 & 38.3 respectively, *P*<0.001, Table S2, Dunn's test *P*<0.05). Metamorphosis rates were 1.6 times faster for *H. sexdentatus* and 1.7 times faster for *L. variegatus* in the Ambient Reef sound treatment than for the other treatments using Pakiri Beach sound.

## Discussion

Previously, the studies on the auditory capabilities and behavioural response thresholds in marine animals have been focused on fishes and mammals [28], [29], [30], [31], [32], [33]. There are only a handful of investigations on the hearing abilities and behavioural response thresholds of larval fishes, and no previously published results that specifically examine the acoustic behavioural response thresholds of crustacean larva [34], [35], [36]. However, there are a small number of studies which have demonstrated that settlement stages of coastal crabs show an attraction and orientation response to underwater reef sound, although the ecological importance or spatial scale over which these behaviours operate have not been identified [13], [14]. Previous studies investigating the auditory capabilities of adult crustaceans have focused on electrophysiological methods [16], [37], [38], [39]. For example, a study by Lovella *et al.* (2005) described both the anatomy of the sensory structures of the statocyst while also providing electrophysiological evidence of sound reception in the adult prawn, *Palaemon serratus*. The statocyst was shown to be sensitive to the motion of water particles displaced by low frequency sounds ranging from 100–3000 Hz [37]. However, some previous behavioural measurements of hearing ability in fishes have shown experimental animals to be more sensitive than observed using the ABR methods, and results of the two experimental approaches are not always consistent [36], [40].

### Behavioural response threshold levels to reef sound

The experiments replaying North Reef sound to three reef-dwelling crab species, found that *H. sexdentatus* exhibited the highest acoustic response threshold (lowest sensitivity) to underwater sound. For megalopae of this species there was a significant reduction in TTM in sound treatments with sound levels of 125 dB re 1 µPa and above when compared with the Silent treatment. *Leptograpsus variegatus* showed the lowest acoustic response threshold (highest sensitivity) to underwater sound, and there was a significant reduction in time to metamorphosis (TTM) in treatments 90 dB re 1 µPa and above (100, 126 and 135 dB) when compared with the Silent treatment. It is possible that the behavioural response threshold in this species could be lower, and therefore the acoustic sensitivity higher than that measured as there was no sound level treatment intermediate to the Lowest sound treatment (90 dB) and the Silent treatment. A greater range of experimental treatment sound levels would provide better resolution in determining behavioural thresholds in any future studies on this species.

Once the response acoustic response threshold had been met (i.e., showing a significant reduction in TTM compared to the Silent treatment), *C. lavauxi* showed a graded response with decreasing TTM to sound levels exceeding this threshold level. For example, *C. lavauxi* showed the greatest reduction in TTM in the 126 dB and 135 dB sound treatments (60 and 68 h respectively), an intermediate response in the 100 dB sound treatment (84 h) and no response in the 90 dB sound treatment (108 h) when compared to the Silent treatment (114 h). This suggests that proximity to the sound source, or settlement habitat, is important in inducing a faster settlement and metamorphosis. Such a graded response could help to ensure that an accelerated settlement rate does not result in metamorphosis being completed before the swimming megalopae reach their settlement destination. The results also suggest that underwater sound as a settlement and metamorphosis cue does not simply trigger a behavioural response but is more likely to be mediating the behavioural and physiological settlement processes by continuous exposure to the sound cue.

The identification of these acoustic behavioural response thresholds also provides the opportunity to broadly estimate the spatial scale at which these acoustic settlement and metamorphosis cues are operating. It would appear that the acoustic cues have the potential to elicit a response at some distance from the settlement habitat given the acoustic behavioural response thresholds determined in this current study, although this response varies markedly among the small number of species examined. *Leptograpsus variegatus* exhibited the lowest response threshold (90 dB) to replayed reef sound which equated to an estimated maximum settlement response distances of approximately 199 or 39.8 km assuming cylindrical or spherical spreading of sound from the reef, respectively. These distances were substantially greater than for both *H. sexdentatus* and *C. lavauxi*. However, the TTM rate in both *H. sexdentatus* and *C. lavauxi* almost doubled compared with lower sound level treatments once the acoustic threshold had been reached, whereas in *L. variegatus* the experiment could not detect a significant change in the TTM rate across the various sound level treatments. These results suggest that there may be different larval settlement strategies among species, with species with high acoustic thresholds relying on much more rapid settlement once in the immediate vicinity of a suitable habitat, whereas species with lower acoustic thresholds may detect a suitable habitat from greater distances, but not accelerate their settlement response to the same degree in order to provide sufficient time to swim toward and locate the settlement habitat. These preliminary data indicate that sound could be acting as a settlement cue over substantial distances in some species, which may only be relatively matched in their scale of influence by some chemical cues (1–4 km) which are dependent on the physical geography of the area, wind direction and tidal state [41]. Precise estimates of the spatial scales at which the chemical and tactile settlement and metamorphosis cues may operate have not been determined as many of these studies are carried out in a laboratory setting in order to be able to control other experimental variables.

The role of active swimming has been described for the pre-settlement larvae of some decapod crustaceans [42], [43], [44]. In four crab species (*Uca uruguayensis*, *Chasmagnathus granulate*, *Cyrtograpsus angulatus* and *Cyrtograpsus altimanus*) megalopal swimming speeds ranged from 1.2 to 20.8 cm s−1, depending on species and size, with the maximum occurring in *C. altimanus* [42]. In an additional study, five of New Zealand crab species the megalopae have been observed to possess maximum sustained swimming speeds (MSSS) ranging between 2.06 and 10.96 cm s^−1^. For all species examined (*Hemigrapsus* sp., *Austrohelice crassa*, *Macrophthalmus hirtipes*, *Cyclograpsus* sp., *Ovalipes catharus*) MSSS exceeded experimental current velocities for at least three hours of each tidal cycle (up to 25 cm s^−1^) (Meder, unpublished data). It was also observed that megalopae of two species were able to swim continuously for a maximum of 36 h, with one species covering a distance of 7 km (Meder, unpublished data). The extent of these sustained swimming abilities strongly suggest that directed movement towards suitable settlement habitats over considerable distances is feasible in brachyuran megalopae provided a guiding cue is available at these distances. The estimated acoustic detection distances measured for megalopae of some species of crab in the current study suggest that an acoustic reef derived settlement cue has the potential to be effective at some distance from the source.

There are also uncertainties about the utility of the settlement response distances estimated in this current study due to the lack of knowledge on the hearing mechanisms used by larval crabs. However, previous laboratory experimental results for crab settlement and metamorphosis are entirely consistent with the results of matching field experiments [17], and similar methods have also been used to estimate response distances in larval fish [29], [45], [46].

### habitat-specific responses to sound

The findings of this study also indicate that brachyuran crab megalopae may require habitat-specific underwater sounds to act as an effective cue for settlement and metamorphosis. For example, the tunnelling mud crab, *Austrohelice crassa*, showed no response to any sound levels of replayed reef sound, whereas all three reef associated crab species that were tested showed a significant decrease in TTM in response to reef sound once their behavioural threshold had been reached. The reef is not a habitat that is used during any part of the life cycle of *A. crassa* [21], [22] so it would be potentially detrimental for this crab to have a settlement and metamorphosis response to reef sound. However, it is possible that larvae of this species of crab may respond to an acoustic cues produced from estuarine habitats where it is normally found [22] or it is also possible that this species does not exhibit a settlement and metamorphosis response to acoustic cues at all.

The results from the experiments using sound recorded from Pakiri Beach revealed that sound level alone does not explain the settlement and metamorphosis response observed in crab megalopae exposed to ambient underwater reef sound. Megalopae of both *H. sexdentatus* and *L. variegatus* showed no significant response to varying levels of sound from an open sandy beach habitat, even when the sound level was at a similar level (less than 1 dB difference) to the ambient sound at their preferred settlement habitat, rocky reef. There was no significant reduction in TTM in any species tested in the treatments with Pakiri Beach sound at 90, 103, 125 dB re 1 µPa, or a Silent treatment, while there was a significant reduction in TTM in the Ambient Reef sound treatment. These results demonstrate that it is the frequency and temporal composition of underwater sound rather than the sound level *per se* that is an important characteristic for the mediation of settlement and metamorphosis in these settlement stage crab larvae. These results corroborate those of a previous study where the megalopae of five species of both temperate and tropical crab showed a significant decrease in TTM, by almost half in some species, when exposed to sound from their optimal settlement habitat compared to two other unfavorable types of habitats [17].

### Conclusions

The current study found that there is considerable variation in the levels of underwater reef sound that initiate settlement and metamorphosis behavioural responses in the megalopae of three species of New Zealand brachyuran crabs. The measured behavioural response thresholds to ambient underwater reef sound enabled estimations of the spatial range that the acoustic settlement and metamorphosis cue could be operating, which was found to extend to many kilometres in some species. It also provides further evidence of settlement stage crabs discriminating among suitable settlement habitats on the basis of the sound emanating from the habitat. Furthermore, it would appear that it is the composition of the underwater sound rather than sound level alone, which is the important characteristic for an effective acoustic settlement cue.

Overall, these results greatly extend the knowledge and ecological context of sound acting as a settlement and metamorphosis cue for the megalopae of coastal crab species. Future research should focus on gaining greater resolution of the behavioural response threshold sound levels of crab species so we can better define the spatial scale over which this important behaviour operates, and its relative importance, especially to other known settlement cues, in ensuring the successful settlement and recruitment of valuable coastal crab species.

## Supporting Information

Table S1 **Comparisons among median TTMs and metamorphosis rates for the North Reef experiments in four crab species.**
(DOC)

Table S2 **Statistical comparisons among median TTMs and metamorphosis rates in Pakiri Beach experiments for four crab species.**
(DOCX)
