# Supplementary material for: Behavioural Response Thresholds in New Zealand Crab Megalopae to Ambient Underwater Sound
Source: PLoS One. 2011 Dec 7;6(12):e28572. doi: 10.1371/journal.pone.0028572 (PMC3233601; doi:10.1371/journal.pone.0028572)
Supplement: Table S1 — Comparisons among median TTMs and metamorphosis rates for the North Reef experiments in four crab species. (DOC) [file pone.0028572.s001.doc]

| **Species** | **Total number of individuals (n)** | **Treatment**  **(sound level dB)** | **Median TTM (h)** | **H – statistic** | ***P* – value** | **Metamorphosis rate** | **F - statistic** | ***P* - value** |
| --- | --- | --- | --- | --- | --- | --- | --- | --- |
| ***Hemigrapsus sexdentatus*** | 24 | High (135) | 48 | 53.9 | ***< 0.001 | 9.4 | 5.8 | ***0.002 |
|  | 24 | Ambient (126) | 48 |  |  | 10.8 |  |  |
|  | 24 | Low (100) | 75 |  |  | 8.3 |  |  |
|  | 24 | Lowest (90)  Silent | 78 |  |  | 5.3 |  |  |
|  | 24 |  | 84 |  |  | 6.1 |  |  |
| ***Cyclograpsus lavauxi*** | 30 | High (135) | 68 | 25.8 | ***< 0.001 | 6.1 | 11.2 | ***0.001 |
|  | 23 | Ambient (126) | 60 |  |  | 5.1 |  |  |
|  | 30 | Low (100) | 84 |  |  | 3.7 |  |  |
|  | 30  27 | Lowest (90)  Silent | 108 |  |  | 3.7 |  |  |
|  |  |  | 114 |  |  | 3.4 |  |  |
| ***Leptograpsus variegatus*** | 15 | High (135) | 96 | 23.8 | ***< 0.001 | 4.8 | 0.5 | 0.742 |
|  | 15 | Ambient (126) | 90 |  |  | 4.4 |  |  |
|  | 15 | Low (100) | 108 |  |  | 4.3 |  |  |
|  | 15 | Lowest (90)  Silent | 108 |  |  | 4.3 |  |  |
|  | 15 |  | 150 |  |  | 3.9 |  |  |
| ***Helice crassa*** | 30 | High (135) | 57 | 6.1 | 0.177 | 6.0 | 0.6 | 0.813 |
|  | 30 | Ambient (126) | 62 |  |  | 5.7 |  |  |
|  | 30 | Low (100) | 73 |  |  | 6.9 |  |  |
|  | 30 | Lowest (90)  Silent | 57 |  |  | 6.1 |  |  |
|  | 30 |  | 71 |  |  | 7.8 |  |  |

**Supporting Information S1**

Table S1: Comparisons among median TTMs and metamorphosis rates for the North Reef experiments in four crab species.

***Asterisks indicate a significant difference in median TTMs between treatments (*P* < 0.05, Kruskal-Wallis test) and significant difference in metamorphosis rate (*P* < 0.05, ANOVA).
