# Supplementary material for: Behavioural Response Thresholds in New Zealand Crab Megalopae to Ambient Underwater Sound
Source: PLoS One. 2011 Dec 7;6(12):e28572. doi: 10.1371/journal.pone.0028572 (PMC3233601; doi:10.1371/journal.pone.0028572)
Supplement: Table S2 — Statistical comparisons among median TTMs and metamorphosis rates in Pakiri Beach experiments for four crab species. (DOCX) [file pone.0028572.s002.docx]

| **Species** | **Total number of individuals (n)** | **Treatment**  **(sound level dB)** | **Median TTM (h)** | **H – statistic** | ***P* – value** | **Metamorphosis rate** | **F - value** | ***P -* value** |
| --- | --- | --- | --- | --- | --- | --- | --- | --- |
| ***Hemigrapsus sexdentatus*** | 15 | High (125) | 60 | 26.8 | ***<0.001 | 8.6 | 32.9 | ***<0.001 |
|  | 15 | Ambient (103) | 54 |  |  | 4.8 |  |  |
|  | 15 | Low (90) | 54 |  |  | 6.5 |  |  |
|  | 15 | Silent  Ambient Reef (126) | 54 |  |  | 8.6 |  |  |
|  | 15 |  | 30 |  |  | 13.7 |  |  |
| ***Leptograpsus variegatus***  ***Asterisks indicate a significant difference in TTMs among treatments (*P* < 0.05, Kruskal-Wallis test) and significant difference in metamorphosis rate (*P* < 0.05, ANOVA). | 15 | High (125) | 84 | 12.8 | ***0.012 | 6.1 | 38.3 | ***<0.001 |
|  | 15 | Ambient (103) | 90 |  |  | 4.7 |  |  |
|  | 15 | Low (90) | 90 |  |  | 5.4 |  |  |
|  | 15 | Silent  Ambient Reef (126) | 84 |  |  | 5.0 |  |  |
|  | 15 |  | 66 |  |  | 10.5 |  |  |

**Supporting Information S2**

Table S1: Comparisons among median TTMs and metamorphosis rates for the North Reef experiments in four crab species.
